# Early breeders choose differently – Refining measures of habitat quality for the yellow-bellied sapsucker (*Sphyrapicus varius*), a keystone species in the mixedwood boreal forest

**DOI:** 10.1371/journal.pone.0203683

**Published:** 2018-09-12

**Authors:** Kelly A. Squires, Fred L. Bunnell

**Affiliations:** 1 School of Resource and Environmental Management, Faculty of Environment, Simon Fraser University, Burnaby, British Columbia, Canada; 2 Faculty of Forestry, University of British Columbia, Vancouver, British Columbia, Canada; Liverpool John Moores University, UNITED KINGDOM

## Abstract

Despite the prevalent use of nest-site selection studies to define habitat quality for birds, many studies relying on use-availability analysis have found poor correlations between selected vegetation and reproductive success. Using 3 years of data from northeastern British Columbia (2007–2009), we determined timing of breeding from hatching dates and contrasted the nest-site selection of earlier (*n* = 22) with later-nesting pairs (*n* = 36) of yellow-bellied sapsuckers (*Sphyrapicus varius*), because early breeders were expected to be more reproductively successful. We then compared these choices with those identified from use-availability analysis, and determined whether reproductive performance (fledgling production) was related to selected vegetation. None of the vegetation characteristics selected for nest sites from available vegetation predicted reproductive performance. Earlier-nesting pairs fledged more young on average than later breeders (4.41, SE = 0.18 versus 3.92, SE = 0.16), and chose less decayed aspen trees for nesting, that were surrounded on average by 3 times the number of food trees (paper birch, *Betula papyrifera*). Potential preference for birch trees was masked in the use-availability analysis, because the selection rate was dominated by the choices of the larger number of later-nesting pairs. Similarly, the majority (69%) of nest cavity entrances faced south, but earlier breeding pairs excavated northward-oriented cavities more frequently than did later breeding pairs, which strongly predicted their higher fledgling production. To our knowledge, our study is the first to compare the choices of early versus later breeders to test the efficacy of use-availability studies in defining habitat quality. We found that use-availability analysis was inadequate for determining vegetation characteristics related to reproductive performance. In contrast, measuring the distinct preferences of earlier breeders resulted in an improved ability to measure habitat quality and explain the spatial distribution of yellow-bellied sapsuckers, a keystone species of the mixedwood boreal forest.

## Introduction

One of the most important yet challenging goals of wildlife management is assessing habitat quality to derive prescriptions for retaining high quality habitat across disturbed landscapes. It can be useful in some management contexts to define high quality habitat as that which produces the highest contribution to population growth per unit area [1, 2]. But habitat preferences appear to be adaptive and heritable, and so the habitat choices of genetically advantaged individuals ultimately drive population dynamics [3]. Thus, defining high quality habitat as habitat that confers the highest contribution to population growth per individual corresponds with the underlying evolutionary determinants of quality [2, 4]. A key focus of avian ecology is to derive management prescriptions to promote high quality habitat based on the preferences of individuals for nest site characteristics, because these features can be strongly related to factors that affect fitness, particularly predation of young [5, 6].

But management prescriptions derived from individual preferences can be ineffective if nest site preferences do not correlate well with fitness. The underlying assumption of the use-availability designs commonly applied in nest-site selection studies is that preference and habitat quality can be measured from the frequency by which certain habitat characteristics or types (i.e. density) are used relative to available choices [2, 7]. This assumption has merit as the main prediction within an ideal free distribution, wherein individuals are free to choose habitat that maximizes their fitness, such that frequently chosen (i.e. high density) habitat is of high quality until saturation [8]. However, it appears to be more the rule than the exception that habitat characteristics correlated with reproduction are different from those that birds frequently select for nest sites from available habitat [9–12]. In a review of 70 studies, avian nest success was incongruent with frequently-selected nest site characteristics in 80% of studies across all species, and in 90% of studies of cavity-nesting birds [12].

Disconnect between nest-site selection and reproductive performance may result from ecological processes, such as 'ecological traps' revealed by low fecundity in areas of high density [13], and also from methodological issues, such as the failure to distinguish preferences of earlier versus later breeders [12]. Under the ideal despotic distribution, preference and thus habitat quality can be measured from the choices of dominant or more experienced individuals that breed earlier and exclude less experienced breeders from preferred habitat [8, 12, 13]. For ideal despotic populations composed of a majority of inexperienced breeders making or being forced to make sub-optimal choices, erroneous definitions of habitat quality will be generated when nest site preferences are measured from use-availability analysis. Under this condition, habitat that is frequently selected compared to available represents the choices of breeding pairs with lower reproductive performance. Indeed, an underlying ideal despotic distribution may be a key reason why habitat selection measured from use-availability analysis is so often incongruent with habitat correlated with high reproductive success, particularly among territorial birds [12].

The first objective of our research was to test whether habitat quantified from an assumed ideal free distribution of breeding yellow-bellied sapsuckers in mixedwood boreal forest of northeastern British Columbia (BC) differed from habitat quantified assuming an ideal despotic distribution. Our second objective was to test whether nest density of yellow-bellied sapsuckers was predicted by the quantity of vegetation attributes they selected for nesting at the scale of a forest patch. Following the assumption of an ideal free distribution, we measured vegetation preferences for nest sites by applying use-availability analysis to identify frequently selected nest site characteristics in comparison to random sites. Following the assumption of an ideal despotic distribution, we contrasted the vegetation choices for nest sites of early with later breeding pairs, because early breeders of this territorial species were expected to exclude later breeders from preferred choices, particularly for high quality trees in which to excavate nesting cavities. Earlier breeders are usually more experienced birds that settle in preferred habitat first and achieve higher reproductive success. Thus, we expected earlier breeders to have settled territories earlier or to have been more experienced or dominant individuals able to exclude later breeders from preferred habitat [13–15]. We tested whether their distinct vegetation choices would predict higher productivity, and thus could be used to define habitat preference and quality. We used fledgling production quantified by video-monitoring of nests as a measure of reproductive performance, while acknowledging that post-fledging measures of reproductive performance may be necessary to adequately represent fitness [16]. As the corollary, we predicted that vegetation characteristics that were frequently selected from the available choices would only predict reproductive performance if the majority of the breeding population were early breeders that were able to settle in preferred habitat.

Sapsuckers have been termed a ‘double’ keystone species because their sap wells and abandoned nest cavities provide both food and refuge for many species [17]. Abandoned cavities excavated in trembling aspen trees (*Populus tremuloides*) by yellow-bellied sapsuckers provide nesting sites for flying squirrels (*Glaucomys sabrinus*,) and most roost sites of big brown bats (*Eptesicus fuscus)* [18, 19]. Sapwells created by sapsuckers are used for food particularly by red squirrels (*Tamiasciurus hudsonicus*), rufous hummingbirds (*Selasphorus rufus*), and bees (*Apidae*) [17, 20, 21]. Deriving management prescriptions for high quality nesting habitat of yellow-bellied sapsuckers is an efficient way to manage for resource needs of the diverse assemblage of species using sap wells and cavities.

We developed *a priori* hypotheses and predictions based on field observations and published studies regarding habitat characteristics that we predicted would influence selection of nest sites and territories, and influence reproductive success. Expecting that yellow-bellied sapsuckers chose nest trees in which large cavities could be easily excavated to provide more space for young [22], we predicted that among available dead and decaying aspen trees, they would choose larger dead trees, and larger more decayed living aspen trees. Yellow-bellied sapsuckers more often choose a different but nearby nesting tree in successive breeding seasons [21]. Thus, we predicted that yellow-bellied sapsuckers would choose nest sites based on the availability of future options for nesting. Pairs that establish territories in areas with higher densities of preferred trees may be ensuring against loss, given the relatively high rates at which aspen trees fall (~50% loss over 15 years) [23, 24]. At the scale of the territory core, we hypothesized that sapsuckers chose to nest where living birch trees (*B*. *papyrifera*) and shrubs were more abundant because studies show these are used as food resources. Studies have documented that adults during nesting frequently feed from and defend sapwells within about 50 m of nest trees and that there is a greater incidence of sapwells in birch trees in boreal forest [21], [25, 26]. Shrub sap is also a food source for adults and young fledglings [21], and shrubs may also confer predator protection. At the territory core scale, we predicted that yellow-bellied sapsuckers would choose to nest in mixedwood forest (20%-80% deciduous trees). Sap from coniferous trees is important to sapsuckers in the early spring, before deciduous sap rises [21, 27]. Thus, we predicted higher selection for mixedwood forest where a mixture of coniferous and deciduous trees provides the most food resources, while also containing an adequate supply of nest trees [27].

We predicted that earlier breeding pairs would show higher selection for 2 cavity characteristics that would predict higher fledgling production—southward orientation of cavity entrances because of higher cavity temperature [28], and cavity height due to lower predation by ground-based predators in higher cavities [29]. We expected that frequently excavated trees represent high quality habitat, because studies have shown benefits to breeding performance from re-use of nesting trees [30]. Thus, we expected earlier breeding pairs to nest more often in trees with multiple cavity holes compared to later breeding pairs, and we predicted a positive relationship between counts of fledglings and other cavity holes in nest trees.

## Methods

### Study area

**Fig 1:**
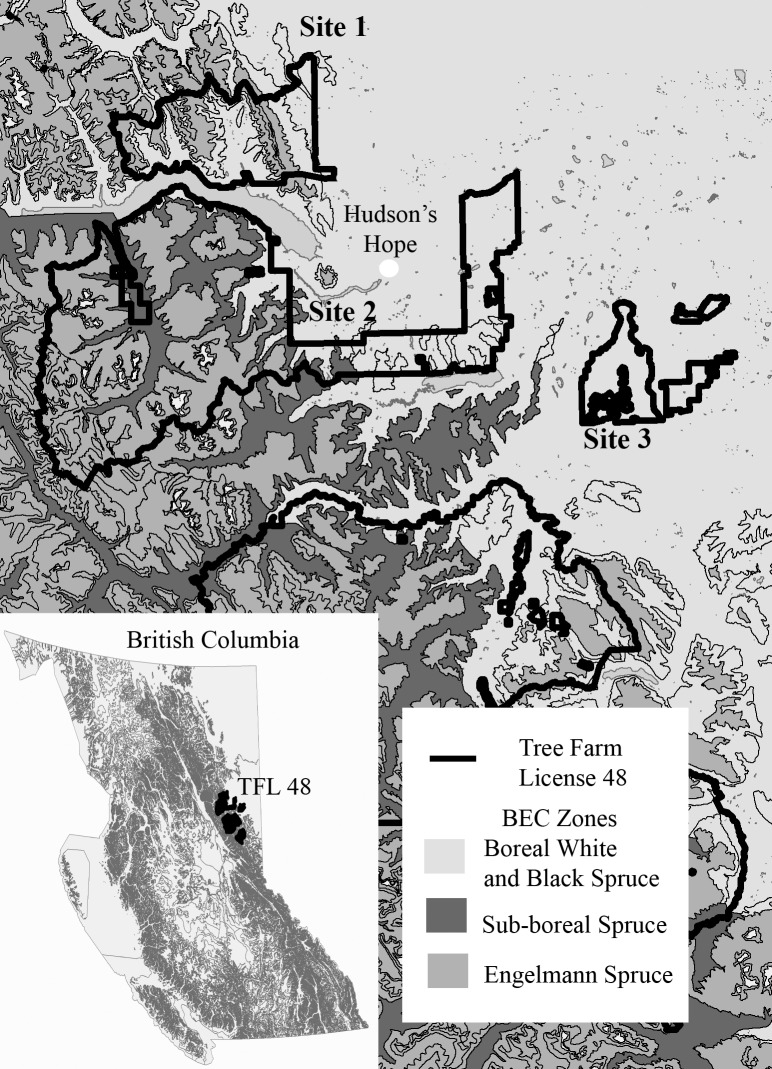
Location of three study sites in northeastern British Columbia, Canada.

This research was conducted from late May to July (2007–2009) in 3 study sites within an area about 80 x 80 km centered on the town of Hudson's Hope (56°1'54.74''N 121°54'18.29''W) within the Boreal White and Black Spruce (BWBS) Biogeoclimatic Zone [31] of northeastern British Columbia (Fig 1). Work was conducted within or adjacent to Tree Farm License 48 (~660,000 ha), which is public land for which no specific permission was required to conduct the research. Clearcut logging of lodgepole pine (*Pinus contorta var*. *latifolia*), white spruce (*Picea glauca*), and trembling aspen has proceeded since 1950 across about half of the study area. The footprints of mining, and oil and gas activities were distributed widely but covered only 1% of the area [32]. Typical of boreal forest, frequent fires have resulted in very few stands older than 180 years. Relative to natural disturbances, logging in the mixedwood boreal forest has resulted in fewer mixedwood stands [33].

One study site (5500 ha) was located at low elevations (600–900 m) in the eastern lee of the Rocky Mountains; the other 2 sites (3000 and 4000 ha) were on the boreal plain extending throughout northeastern BC (600–750 m). All sites were in managed forest characterized by a mosaic of unlogged patches of forest (90–180 years old, 12–60 ha) differing in the ratio of deciduous to coniferous trees ("forest composition"), interspersed with regenerating clearcuts of varying ages (~ 40–80 ha) and criss-crossed by seismic lines. Most unlogged stands including those sampled were likely to have been influenced by human-created openings (clearcuts, logging roads, seismic lines). Deciduous forest patches were dominated by trembling aspen with lesser amounts of balsam poplar (*P*. *balsamifera*), paper birch (*Betula papyrifera*) and Alaska birch (*B*. *neoalaskana*), mixedwood patches dominated by a mixture of aspen and white spruce, and coniferous patches dominated by white spruce and lodgepole pine. In all forest types, willow (*Salix* spp), Sitka alder (*Alnus sinuata*), and green alder (*A*. *viridis*) were the only tall (> 1.3 m) shrub species present, though tall shrubs were infrequent in coniferous stands.

### Measuring nesting habitat assuming an ideal free distribution

We obtained the necessary approval to conduct all aspects of this field research from the Animal Care Committee of Simon Fraser University under the guidelines of the Canadian Council on Animal Care. This work did not involve endangered or protected species. All data are presented as means ± SE unless otherwise stated. We monitored nests using standardized protocols [34] in patches of old unlogged forest (100–180 years) that varied in forest composition (ratio of deciduous to coniferous trees). We found nests by following parents or the sound of begging nestlings to the nest, or by finding fresh wood chips at the base of trees. Most nests (65%) were found by walking transects in forest patches surveyed to estimate nest density. The other 35% of nests were found while travelling in surrounding old forest patches too small for transects. Yellow-bellied sapsuckers and vegetation were sampled within the majority of old unlogged forest within each study site that was accessible by logging roads.

Under the assumption of an ideal free distribution, we measured nesting habitat by applying a use-availability design. We quantified the odds of selecting vegetation variables measured at 3 spatial scales by pairing nest sites (n = 56) with available but unused (control) sites within a ‘constrained’ study design [35]. The 3 spatial scales were the nest tree, the nest site, and the territory core. The radius of the nest site was chosen as the radius of a standard vegetation plot (11.3 m), which matches the scale at which pairs strongly defend nest sites [21]. Because average inter-nest distance was 135 m, we chose to delineate the territory core with a 41.3 m radius to ensure non-overlapping vegetation sampling among neighbouring territories. We chose available, unused trees by walking to a pre-selected coordinate (see details below) using a handheld GPS, and selecting the randomly-chosen *n*^th^ tree (range 1–12 trees) within a 30-m belt transect along a random direction from the coordinate. Within a GIS database, coordinates were chosen in stands 100–180 years old at a randomly chosen direction and distance between 250 and 350 m (average 300 m) from the nest tree coordinate. This distance was used to quantify selection at the territory scale, based on the assumption that average territory size was not more than 7 ha [21, 27]. The presence of internal rot in live decaying and dead aspen trees indicates availability for cavity construction [36]. Inclusion criteria for available trees were: dead or live decaying aspen trees with at least one fungal conk. Aspen trees in advanced stages of decay can be identified by the presence of conks on the stem, which are the fruiting bodies of the *Phellinus tremulae* fungus that causes internal decay [36, 37]. We assumed that one fungal conk indicated sufficient decay for cavity excavation, based on the observation that decay extends 1 m below and 3 m above each conk on average [37]. The presence of just one conk usually indicates extensive decay, since aspen trees already contain on average 40% decay prior to forming conks [38].

We confirmed that all available random sites were not used after not finding nest cavities or sapwells, and by eliciting no responses to playback of sapsucker calls and drumming during the pre-fledging period in June. All vegetation variables were measured during the post-fledgling period in late July; none of the variables were expected to measurably change between the nesting and post-fledgling periods. We were able to measure breeding productivity on 58 territories out of a total of 120 territories found. On the remaining 62 territories, nests were found during the late nestling stage which may have biased results toward successful nests, or cavities were too high for video monitoring (44% of unmonitored nests were above the maximum height of 13.7 m that could be monitored). Of the 58 territories for which we gathered data on breeding phenology and productivity, we were able to find paired unused sites for 50 territories. Each unused site was centred on a live decaying aspen tree with at least one fungal conk and showed no signs of use by yellow-bellied sapsuckers. An additional 6 territories that we found early in the season but were not able to video monitor were added to the use-availability analysis, resulting in sample sizes of 58 yellow-bellied sapsucker pairs for analyses related to breeding and 56 territories to compare to unused sites.

### Measuring nesting habitat assuming an ideal despotic distribution

We measured nesting habitat under the assumption of an ideal despotic distribution by first determining whether earlier breeding pairs selected different nest site characteristics than pairs that bred later, and then by determining whether breeding productivity was related to their distinct choices. We used hatching date as a measure of breeding phenology. For most pairs (40 of *n* = 58), we estimated hatch date by using a video camera mounted on an extendable pole ('TreeTop Peeper', Sandpiper Technologies Inc., Manteca, California). Nests were video monitored during the incubation and early hatchling stages. The remainder of nests were found during the middle of the hatchling stage; for these nests we estimated hatch date as 25 days earlier than the estimated fledging date [21]. It is possible that video monitoring may have affected nest attendance with subsequent effects on breeding phenology or productivity. Though an observer effect explained lower probability of nest success of the Lewis’s woodpecker on days when cavities were observed with a video camera, the overall effect was concluded to be minimal given that nests were checked less than four times in total [39]. More intrusive nest monitoring involving extraction of Northern flicker (*Colaptes auratus*) eggs and nestlings through a hole cut in nest trees was found to have no effect on cavity-reuse, though other effects on breeding were not measured [28].

We categorized pairs as 'earlier' breeders (*n* = 22) if their eggs hatched prior to the median of dates (15 June), which differed by only 3 days across years. It is possible but unlikely that 'later' breeders were incorrectly classified because they had failed and re-nested. None of the 30 of 58 nests that we found in the egg stage failed, and re-nesting at these latitudes is infrequent [40].

We measured reproductive success per yellow-bellied sapsucker pair in each year as the number of nestlings within one week of fledging (termed 'fledglings'), which were counted with video monitoring. We assumed that females did not produce more than one brood per season, and that all monitored nests were the only nest attempt by each female [21,40]. Nests were monitored every 3–7 days to record nest stage, to count the number of eggs or chicks, to assess the age of nestlings and estimate hatching and fledging date, evidence of predation, and nest fate (success or failure). Yellow-bellied sapsuckers often re-pair with the same mate and use the same territory in successive breeding seasons [21]. The proximity of nests across successive years was used to delineate territories; nest trees used successively on a territory were relatively close together (23 ± 3.1 m), whereas nest trees on adjacent territories were on average 5 times further apart (135 ± 7.6 m). We were not able to determine if the same pairs returned because we did not uniquely mark birds, so we randomly chose one year of data from 7 territories with multiple years of fledgling counts.

### Relating nest density to selected vegetation

We determined whether the density of yellow-bellied sapsucker nests could be predicted from the density of vegetation they selected for nesting. We applied a stratified sampling design by classifying polygons of old forest (‘patches’) into increments of 20% deciduous trees with a GIS database using the British Columbia Vegetation Resources Inventory (VRI). Nests were counted within 87 forest patches (16–21 patches per 20% increment of deciduous trees) within 100 m of the center line of 107 200-m transects walked perpendicular from the edge of logging roads (~30 minutes per transect). Transects were positioned so that the area surveyed represented a 4-ha square plot placed with 100 m between the plot and patch edges. Most patches (*n* = 67) were too small to contain more than one 4 ha plot; the remainder (*n* = 20) were surveyed with 2 adjacent plots. Transect surveys were conducted by the same observer across years and started in late May when most pairs had initiated egg laying and continued until 30 June. We conducted 2 surveys at least 7 days apart on each transect per year, once in the first half (prior to 15 June) and once in the second half of the breeding season.

Nests in the late nestling stage may be more detectable to observers because older nestlings vocalize loudly [41]. Nests found between egg-laying and the mid-hatchling stage were on average only 6 m further from transects than nests found in the late nestling stage (42 ± 5.0 m, n = 38 versus 36 ± 4.8 m, n = 27). However, nest counts declined with distance from the transect. We adjusted for non-detectability of nests further from the transect by fitting a half-normal detection function truncated at 100 m to the perpendicular distances from nests to transect lines [42]. We estimated nest density per forest patch as the total number of nests adjusted for detectability per plot (4.0 ha), averaging nest counts from patches surveyed with 2 transects.

To relate nest density to densities in forest patches of vegetation selected at nest sites, the same vegetation characteristics measured around nests were measured and averaged across 4 plots centered on the end of line transects within the 87 patches. One plot was centered on the end point, and 3 additional plots were positioned 30 m away along directions separated by 120^0^. We visually estimated forest composition (% deciduous trees that reached the canopy) within forest patches as the average of estimates out to about 30 m at 25-m intervals along the 200-m line transects surveyed for nests. We also visually estimated forest composition to about 30 m from the center of the four 11.3 m radius vegetation plots centered on the end of line transects. We used the average of the visual estimates along and at the end of line transects in statistical analyses relating nest density to forest composition. Visual estimates were highly correlated (*r* = 0.92) with estimates based on basal area derived from the diameter at breast height (dbh) of all trees (dbh > 15 cm) within vegetation plots.

### Habitat variables in models

Using field observations and published studies, we developed *a priori* models of vegetation characteristics representing our hypotheses about habitat characteristics that we predicted would influence selection of nest sites and territories, and influence reproductive success (Table 1). We used the same habitat variables to test hypotheses under an assumed ideal despotic distribution as an assumed ideal free distribution (Table 1). However, instead of predicting higher amounts of habitat variables at nest sites compared to unused sites (ideal free), we predicted higher amounts of the same habitat variables at the nest sites of earlier compared to later breeding pairs, and a positive relationship between habitat variables and fledgling production (ideal despotic).

**Table 1:** Hypotheses and variables used to construct candidate models to test predictions about yellow-bellied sapsucker nest-site selection and reproductive success in northeastern British Columbia, 2007–2009. Except for a quadratic relationship to % deciduous and tree diameter, all predictions are for positive linear relationships between the responses of breeding pairs and habitat resources.

Hypothesis	Variable
Large trees are selected to provide opportunities to excavate large cavities that provide more space for young	Diameter of live decaying aspen trees
More decayed aspen trees are selected because these are easier to excavate and offer more opportunities to excavate large cavities	Count of fungal conks on aspen trees
Nest trees are selected near other potential nest trees to provide future options for cavity excavation	Density of live decaying aspen trees at the nest site and territory core scales
Nest sites are selected near birch trees to provide a nearby and thus easily defended food source (sap) for adults and young	Density of living birch trees at the territory core scale
Nest sites near shrubs provide a nearby food source (sap and insects) for adults and young and predator protection	Percent cover of shrubs at the territory core scale
Mixedwood forest is selected for nesting because a mixture of coniferous and deciduous trees provides more abundant resources (e.g. earlier availability of tree sap)	Forest composition (% deciduous trees) within nesting territories. Selection for mixedwood supported by quadratic relationship (% deciduous^2^)
Frequently excavated nesting trees are high quality trees	Count of other cavity holes on nest trees
Cavities with entrances oriented southward provide beneficially warmer cavities	Orientation of cavity entrance
Higher cavities provide protection from ground-based predators	Cavity entrance height

We tested our prediction that yellow-bellied sapsuckers would choose to nest in large easily-excavated trees using diameter at breast height dbh as a correlate of tree size, and the number of fungal conks as a reliable correlate of the amount of internal decay and thus excavation ease [37].

At the nest site scale, we tested our prediction that pairs would choose to nest in areas with higher densities of preferred trees by using the density of live decaying and dead aspen trees of the same size range selected by sapsuckers in our study (22–52 cm dbh), measured within one 11.3 m radius circular plot centered on nest and available non-nest trees. We also tested this prediction at the territory core scale, which was measured within the nest site plot, and within 3 additional 11.3 m radius plots positioned 30 m away along directions separated by 120^0^. The location of the first outer plot was chosen randomly.

At the scale of the territory core, we hypothesized that sapsuckers chose to nest where food resources were more abundant (Table 1). As correlates of food abundance, we measured the density of living birch (*B*. *papyrifera*) stems > 15 cm dbh visually estimated the % cover of shrubs taller than 1.3 m (*Alnus* and *Salix* spp.) as the percent of ground area covered when the shrub crowns were projected vertically, excluding any overlap. To test our prediction that pairs would choose to nest in mixedwood forest, we estimated forest composition (% deciduous) as the average basal area of deciduous trees (> 15 cm dbh) among the four 11.3 m radius plots within 41.3 m of nest and unused trees. We counted the number of other cavity holes in nest trees to test our prediction that frequently excavated trees represent high quality habitat. To test our prediction that sapsuckers would excavate higher and south-oriented cavities, we measured cavity height from the ground to the nearest 0.1 m with a clinometer, and we categorized the orientation of cavity entrances as south (90°–225°) or north (270°–45°).

### Statistical analyses

To compare predictions under the ideal free and ideal despotic hypotheses, we conducted 3 separate statistical analyses using R Program (R Version 3.0.0, www.r-project.org, accessed 20 June 2013). We used conditional logistic regression to estimate the odds of selecting vegetation for nesting relative to vegetation at available unused sites [35]. We compared the vegetation choices of earlier versus later breeding pairs using binomial generalized linear modeling (GLM) with a log link. We related groupings of low (0–3), moderate (4), and high (5–6) fledgling production to vegetation using ordinal logistic regression. Fledgling counts were under-dispersed (variance < mean), and thus generalized linear modeling with a Poisson error distribution yielded unreliably large standard errors, resulting in an inability to identify relevant relationships in our first analysis of these data [43, 44, 45]. We also used GLM of fledgling counts from 0 to 6 with the Conway-Maxwell Poisson error distribution [45], but this analysis yielded the same results as the ordinal logistic regression, so we reported the ordinal results only.

Because of the patchy distribution of aspen in mixedwood forests, the nest sites of yellow-bellied sapsuckers may be spatially clustered leading to non-independent model residuals and thus inaccurate estimates for the standard errors of model parameters. We tested for and found no evidence of spatial auto-correlation using Moran’s I in the Pearson residuals of the use-availability model (P = 0.34) and of the logistic regression model comparing the nest site characteristics of earlier with later breeding pairs (P = 0.29).

Based on our hypotheses, we constructed *a priori* models based on combinations of variables for habitat resources at different scales (Table 1). Candidate sets included a relatively small number of models to avoid spurious results [46, 47]. We included year, study site, and hatching date among models in the candidate set testing predictions related to fledgling production. All variables were uncorrelated (*r* < 0.40), except the density of aspen trees at the nest and territory scales (*r* > 0.60). To avoid collinearity, we did not include these two variables in the same model. The candidate set of models included a quadratic term for the proportion of deciduous (% deciduous^2^) to test our prediction that sapsuckers would choose to nest in patches of mixed wood forest. We also included a quadratic term for tree size (dbh^2^) to compare support for our prediction that sapsuckers would choose to nest in large aspen trees, with the alternative prediction that they would choose to nest in intermediate-sized trees. We compared 32 models for the use-availability design, and 52 models for the analysis comparing the choices of earlier to later breeders. We then included the best models from each analysis in the candidate set of 30 models of fledgling counts to test whether selected vegetation predicts fledgling production.

Within the 87 surveyed patches, we tested whether the density of sapsucker nests was related to patch-scale densities of vegetation that sapsuckers chose for nesting. We used GLM with a log link and Poisson error distribution, and chose among a candidate set containing all combinations of selected vegetation variables, their interactions, and % deciduous and % deciduous^2^ (i.e. mixedwood patches) to control for possible effects of forest composition.

Within an information-theoretic approach, evidence of support for models were compared among models in candidate sets using Akaike's Information Criterion corrected for small sample sizes (AICc) [47]. The best approximating model was identified as that with the lowest AICc. Model weights (*w*) were computed from the differences in AICc of each model compared to the AICc of the most likely model [47]. We computed model weights across all models, though we acknowledge that this distributed weight to non-competitive models differing from best models by 2–4 ΔAICc with only the addition of uninformative variables [48]. Models with ΔAICc < 2.0 from the best model were considered to be competitive, but only if these models contained different variables or if variables added to the best model improved log likelihood. We did not average model coefficients in cases of model uncertainty, and instead reported parameter estimates and standard errors from the best models [49]. We reported 85% confidence intervals so that inferences were compatible with using AICc for model comparison [48].

## Results

We found 178 active nests in 163 trees on 120 breeding territories during the 3 years of this study. Except for one nest in a poplar snag, all nest cavities had been excavated in aspen trees with internal rot (91% in live decaying, 9% in dead aspen), as evidenced by fungal conks on 97% of trunks. Cavity entrances were on average 10.7 m (± 0.38) high, while average height of monitored nests was 7.9 m (± 0.35). The same proportion of unmonitored as monitored cavities had south-facing entrances (67% and 69%).

### Nesting habitat assuming an ideal free distribution

Model selection based on AICc yielded 2 competing models of a total of 32 in the candidate set of models comparing used with available nest sites (S1 Table, Cox-Snell pseudo R-squared goodness of fit of best model = 0.40 of 0.50). Yellow-bellied sapsuckers excavated nests (n = 56) in trees that were on average 8.1 ± 1.3 cm smaller and intermediate in size with twice as many fungal conks (4.8 ± 0.9) and surrounded by twice as many intermediate sized live decaying aspen trees within 0.04 ha (1.9 ± 0.6) compared to available unused trees (Table 2). These results do not support our prediction that sapsuckers would choose large trees to provide more space for their young, but do support our predictions that pairs would choose easily excavated trees with extensive internal rot in sites with multiple potential nest trees to secure future nesting options. Compared to unused stands, yellow-bellied sapsuckers did not show selection for mixedwood stands as predicted. Probabilities were low for all other models, and thus we found no support for the hypothesis that yellow-bellied sapsuckers chose nest trees based on food availability within the nest site or territory core, or the density of potential nest trees at a larger spatial scale (within 41.3 m) than the nest site.

**Table 2:** Parameter estimates, odds ratios, and confidence intervals (CI) from the most supported conditional logistic regression models relating yellow-bellied sapsucker nest-site selection in comparison to available unused sites in northeastern British Columbia, 2007–2009 (*n* = 56 pairs of sites).

Variable	Sample mean (±SE)		Parameter estimate	Odds ratio	Odds ratio 85% CI
	Nest	Unused			
Dbh^a^	35.3 (0.67)	43.4 (1.16)	1.22	3.39	1.19–9.58
Dbh^2^			−0.02	0.98	0.97–0.99
Conks	9.0 (0.8)	4.2 (0.5)	0.27	1.32	1.05–1.59
Aspen^b^	3.5 (0.5)	1.6 (0.3)	0.27	1.31	1.04–1.61

^a^Diameter at breast height

^b^Count per 0.04 ha of live decaying aspen 22–52 cm dbh

### Nesting habitat assuming an ideal despotic distribution

We measured productivity as the number of fledglings in 65 nest cavities on 58 territories (>1 year of productivity for 7 territories). Nesting success was 98.5% (*n* = 65 nests). Most mortality in successful nests occurred during the egg or early hatchling stage (29 eggs or hatchlings of 155 eggs laid). Mortality of nestlings older than 4 days was observed in only 5 nests (16% of all nest mortality). Average egg success was 83% (range 17–100%, *n* = 30 nests found in egg stage). As defined by hatching date, early breeders compared to later breeders laid more eggs (5.71 ± 0.18, *n* = 7 range 5–6 versus 5.00 ± 0.21, n = 23, range 4–7; Mann Whitney U = 515, P = 0.04) and fledged more young (4.41 ± 0.18, *n* = 22, range 3–5 versus 3.92 ± 0.16, *n* = 36, range 1–6; Mann Whitney U = 115, P = 0.08).

**Fig 2:**
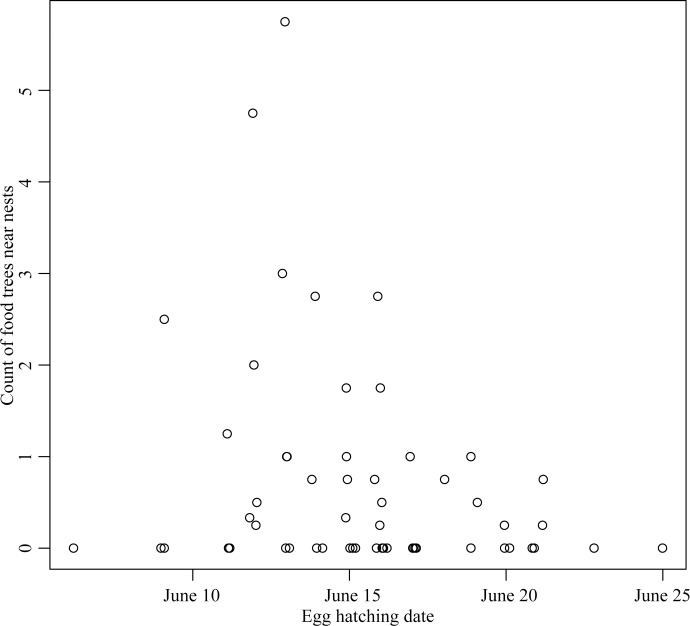
Earlier breeding pairs of yellow-bellied sapsuckers (egg hatching < June 15) nested near (< 41 m) abundant food in the form of sap from birch trees (average tree count in four 0.04 ha plots).

Of a candidate set of 52 models of the nest site choices of earlier versus later-nesting pairs, 3 competitive models were selected using AICc (S2 Table, Nagelkerke's pseudo R-squared goodness of fit for best model = 34.9%). Shrub density was included in the third ranked model, but as an uninformative parameter (odds ratio: 1.15 (0.94–1.40 85% CI). Nest sites of earlier breeders were surrounded on average by 3 times more live birch trees measured within 41 m (Table 3, Fig 2). Most earlier breeding pairs (60% of 22) nested within 41 m of live birch trees, whereas only 44% of later breeding pairs (n = 36) did so. Earlier breeding pairs excavated cavities with entrance holes 1.2 ± 0.5 m higher on average, and more often chose to excavate cavities with hole entrances that faced north (50% earlier versus 19% later breeding pairs excavated north-facing cavities, Table 3). Though all pairs chose trees with more fungal conks compared to available unused trees (Table 2), earlier nesters chose trees with on average 3.5 fewer fungal conks than did later-nesting pairs (Table 3).

**Table 3:** Parameter estimates, odds ratios, and confidence intervals (CI) from the most likely logistic regression models contrasting the vegetation choices of earlier versus later breeding yellow-bellied sapsuckers pairs (*n* = 58) in northeastern British Columbia, 2007–2009.

Variable	Sample mean (±SE)			Parameter estimate	Odds ratio	Odds ratio 85% CI
	Earlier	Later				
Birch^a^	1.2 (0.3)	0.4 (0.1)	0.61		1.84	1.17–2.89
Cavity height	8.7 (0.4)	7.5 (0.4)	0.23		1.25	1.04–1.51
Conks	6.8 (0.8)	10.3 (1.2)	−0.13		0.86	0.41–0.97
Cavity aspect^b^	50%^c^	19%	1.16		2.97	1.21–8.33

^a^Average count of living birch trees measured in 4 plots (0.04 ha) centered on and < 41 m from nest trees.

^b^North versus south.

^c^Proportion of nests facing north.

**Fig 3:**
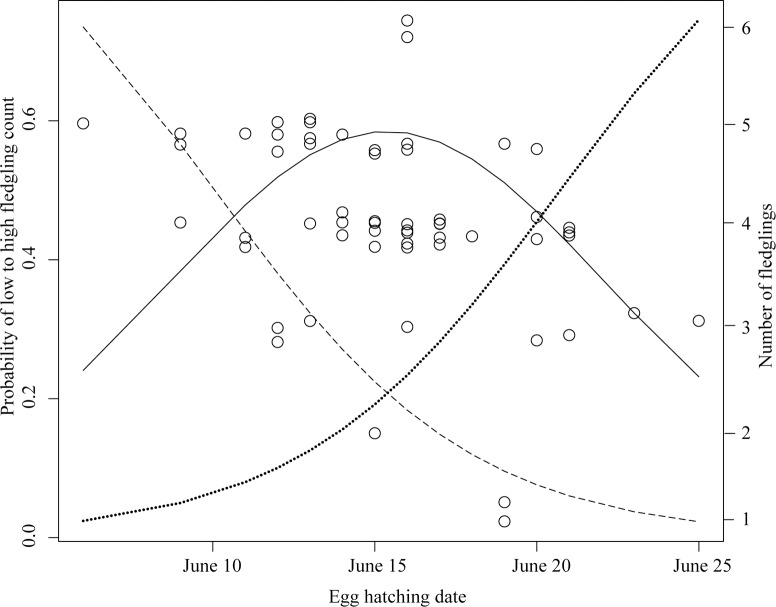
Predicted probability and observed count of yellow-bellied sapsucker fledglings in response to timing of breeding. Predicted probability from ordinal logistic regression of ----- 5–6 fledglings ^____^ 4 fledglings ..... 1–3 fledglings.

Only one model was supported of the candidate set of 30 ordinal logistic regression models relating fledgling production to location, time, and habitat variables (S3 Table). The supported model predicts higher fledgling production with earlier hatching date (Fig 3), and predicts the odds of more fledglings are 5 times higher in north- versus south- facing cavities (Table 4). Cavity height was included in the supported model, but the 85% confidence interval overlaps 1 (odds ratio: 0.86 (0.74–1.02, 85% CI)).

**Table 4:** Parameter estimates, odds ratios, and confidence intervals (CI) of the most supported ordinal logistic regression model relating yellow-bellied sapsucker fledgling production (*n* = 58 pairs) to timing of breeding and cavity characteristics in northeastern British Columbia, 2007–2009.

	Model estimates		
Variable	Parameter estimate	Odds ratio	Odds ratio 85% CI
Hatching date	−0.25	0.78	0.69–0.88
Cavity aspect^a^	1.65^a^	5.21	2.05–13.2

^a^ North versus reference south.

### Predicting nest density from selected vegetation

We tested whether nest density could be predicted from the densities of the vegetation attributes that sapsuckers selected at nest sites within forest patches. By comparing nest sites to available unused sites, we identified intermediate-sized live decaying aspen trees (22 to 41 cm dbh) as a frequently selected characteristic of nest trees. This range encompassed the nest tree size selections made by 91% of earlier and 86% of later breeding pairs. We also identified selection for food trees (live birch) at nest sites, based on the higher selectivity for birch by earlier breeders (Table 3, Fig 2). The candidate set of models included all combinations of the variables % deciduous and % deciduous^2^, density of intermediate-sized aspen trees and live birch trees, and an interaction between aspen and birch. The best supported model predicted the nest density of yellow-bellied sapsuckers from % deciduous^2^, therefore predicting higher nest densities in patches of mixedwood forest (relative rate % deciduous = 1.08 (1.04–1.11 85% CI), relative rate % deciduous^2^ = −0.9994 (0.9991–0.9997 85% CI), Nagelkerke's pseudo R squared = 45%, S4 Table).

**Fig 4:**
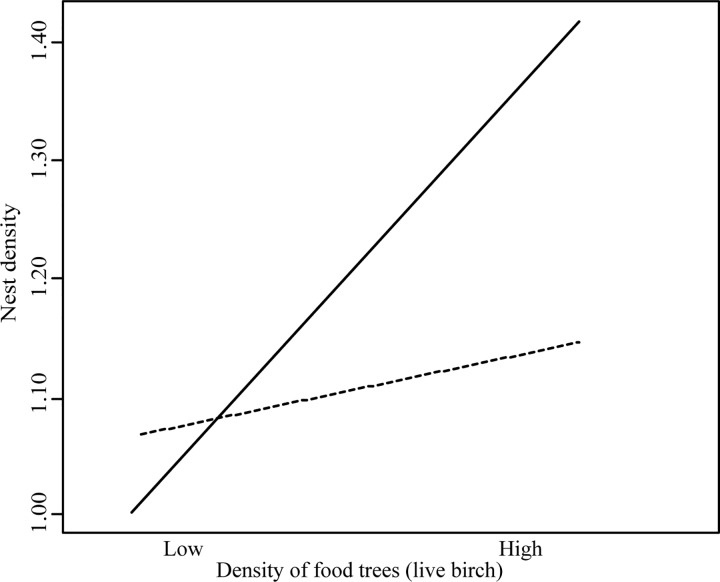
Predicted interaction of the densities of aspen trees used for nesting and food trees (live birch) on nest density of yellow-bellied sapsuckers. High ^____^ and low ----- densities per 0.04 ha of intermediate-sized (22–41 cm dbh) live decaying aspen trees. High and low densities of aspen and birch were set to 75^th^ and 25^th^ percentiles respectively. The other predictor in the model (% deciduous) was held constant at the average.

Fitting a detection function to distances from nests to transect lines resulted in an estimated detection probability of 0.72 (±0.08 SE). Nest density adjusted for detectability in mixedwood patches (20% and 80%) was 9.6 times higher on average than in coniferous-dominated stands (0–20% deciduous trees) where only 2 nests were found, and 1.4 times higher than in deciduous-dominated patches above 80% deciduous trees (average nest density per ha in coniferous-dominated patches: 0.03 ± 0.02, *n* = 20, mixedwood: 0.29 ± 0.05, *n* = 48, deciduous-dominated: 0.21 ± 0.07, *n* = 19). The best model contained an informative interaction between intermediate-sized aspen and living birch (relative rate interaction = 1.26 (1.09–1.47 85% CI)), and was 3 times more likely than the model without the interaction (S4 Table). In forest patches with high densities (75^th^ percentile) of the type of trees that sapsuckers frequently chose for nesting (> 1.4/0.04 ha of 22–41cm dbh live decaying aspen trees), nest density was predicted to increase by 0.26 nests per ha in response to more food (live birch) trees (Fig 4). In contrast, in patches with lowest densities (0.125/0.04 ha) of this type of aspen tree, nest density was predicted to increase by only 0.05 nests per ha in response to birch.

## Discussion

Our results highlight an important reason why researchers have more often failed to find congruence between nest-site selection distinguished from use-availability studies and reproductive performance [3, 12]. We found that frequently selected vegetation identified from use-availability analysis based on an assumed ideal free distribution did not reflect the vegetation choices of the more reproductively successful portion of the population. Habitat variables that best explained fledgling production differed from variables that differentiated nest sites from available sites. In our study, all but one pair fledged at least one young, which is consistent with high nest success rates of cavity excavating species [50], and of yellow-bellied sapsuckers in mature forests in Ontario [40]. But most of the breeding population was composed of pairs that nested later and fledged 0.49 ± 0.24 fewer young on average. Measures of use versus availability were biased to the choices of less successful pairs, because these comprised the majority of the breeding population. To our knowledge, our study is the first to compare the choices of early versus later breeders to test the efficacy of use-availability studies in defining habitat quality [12].

As we predicted, none of the frequently selected characteristics were related to reproductive performance. Instead, we found support for our prediction under an assumed ideal despotic distribution that earlier breeding pairs chose different characteristics for nest sites than later breeding pairs. We found that the more frequent choice to excavate northward-oriented cavities by early compared to later breeding pairs (50% versus 19%) strongly predicted their higher fledgling production. Earlier breeders that excavated north-facing cavities produced 0.8 ± 0.26 more fledglings compared to those that excavated south-facing cavities. Earlier breeders excavated 1.2 m higher cavities on average, but this difference was likely because they excavated more cavities with northward entrances, which among both earlier and later breeders were on average 1.6 ± 0.6 m higher than southward-oriented cavities. All but one of the 11 pairs that performed poorly (3 or fewer fledglings) excavated south-facing cavities. Poor performance was due to higher mortality of eggs and young and not smaller clutches in south-facing cavities (north: 5.4 ± 0.3 eggs (n = 9), south:5.1 ± 0.2 eggs (n = 21)). Most mortality (84%) in our study occurred early, when featherless young were not able to thermoregulate or defend themselves from predators like deer mice (*Peromyscus maniculatus*) [50]. The cavity orientation effects we found do not support the alternative explanations that mortality resulted from disease or nest parasites [51], or from non-viable eggs or nestling starvation due to low food availability [27].

For cavity-nesting birds, the effects of cavity orientation on reproductive success are poorly known [52]. South-oriented cavities are warmer, but tests for both positive and negative effects of cavity temperature on productivity have revealed weak or no effects [28, 52]. Higher cavity temperatures have been hypothesized to promote productivity via energy-efficiencies experienced by adults and young. However, a test of this hypothesis showed a weak positive correlation of cavity temperature with clutch size of northern flickers (*Colaptes auratus*), but no correlation with hatching success or fledgling production [28]. Though [51] found lower body condition of great tit (*Parus major*) nestlings in warmer south-oriented nest boxes, this was not due to a hypothesized positive effect of warmth on parasite loading. Our results suggest other factors than cavity orientation *per se* influenced productivity of yellow-bellied sapsuckers. Though almost all low productivity resulted from south-facing cavities, most pairs that excavated south-facing cavities (30 of 40) performed relatively well (4–6 fledglings).

In contrast to weak or inconsistent effects of cavity orientation, the literature shows consistent effects of cavity shape on woodpecker productivity. Eggs and nestlings in smaller, shallower cavities have been found to be more exposed to temperature extremes and predation [22, 28, 53]. We propose that yellow-bellied sapsuckers drilling from the north side of aspen trees in our study may have accessed relatively large decay compartments, thereby excavated larger or deeper cavities that protected eggs and young from predators or temperature extremes. It seems plausible that north-oriented cavities are less common globally within northern latitudes (31% in our study) because excavation is more difficult from the north, given that sapwood is on average 2 cm thicker on the north side of aspen trees [36, 52]. The ubiquity of woodpecker 'test holes' provides evidence that woodpeckers often encounter reasons to abandon excavation [36, 54]. Because of the southward orientation of decay in aspen tree trunks, excavators may more often abandon excavation from the north side of aspen trees, but more often access large decay compartments when they continue drilling. Perhaps earlier breeding pairs had more time to excavate from the north side of aspen trees, and therefore more often accessed large decay compartments.

Earlier breeding pairs chose to nest near 3 times more food trees (live birch) on average and to nest in less decayed aspen trees. Though neither tree size and decay nor birch density were related to fledgling production, the distinct choices of earlier breeders for more birch trees and less decayed aspen trees likely contribute to long-term reproductive success. Birch sap is an important food resource for adults during breeding. Nesting near live birch may facilitate easier defence of sapwells, which adults defend against other pairs and species, and may confer higher survival for juveniles, because young remain on breeding territories after fledging [21, 25, 55]. By choosing less decayed aspen trees more resilient to trunk breakage, earlier breeding pairs were able to secure trees that provided higher assurance against nest loss [23, 24].

Our finding that both earlier and later breeding pairs chose nest sites with twice the number of suitable nest trees compared to random unused sites supports our hypothesis that yellow-bellied sapsuckers were also motivated to secure territories with multiple options for nesting, thereby providing further assurance against nest loss. Our use of a paired design reveals this as a choice made by yellow-bellied sapsuckers, rather than simply an artefact of the typically clumped distribution of aspen trees, thereby solving a problem identified by other researchers finding a propensity of woodpeckers to nest in patches of aspen trees [56]. Both earlier and later breeding pairs selected intermediate-sized aspen trees, contrary to our prediction that they would select the largest trees in which to excavate large cavities. The same result for yellow-bellied sapsuckers nesting in aspen trees north of our study area was hypothesized to be related to excavation ease, because smaller diameter trees may have thinner sapwood [26, 36]. It is also possible that large trees with sufficient internal decay were less available, perhaps because resistance to fungal infection was the main reason aspen trees were able to grow large.

Contrary to our prediction that frequently excavated trees represented high quality nesting trees, earlier breeders did not select trees with more cavity holes compared to later breeders, and the number of other holes was not related to fledgling production. Our prediction arose from studies showing that tree and cavity re-use in woodpecker species is correlated with higher breeding performance [30, 57]. In our study, most nest sites (56 of 73, including unmonitored nests) were re-used in at least one other year, and 25% of nest trees were re-used (6% cavity re-use). Similar to the Lewis’s woodpeckers (*Melanerpes lewis*) [39], we found that most monitored pairs that re-used nesting trees were earlier breeders (7 of 10), suggesting that re-used trees were higher quality. However, it is possible that trees re-used for nesting were not frequently excavated, as some trees that were beneficially re-used for nesting may have contained no hole if cavities were re-used or just one other hole, whereas trees with many holes may not have been re-used. Because woodpeckers excavate ‘test holes’ and unfinished cavities [30], it is possible that frequent excavation reveals poorer quality trees in which pairs excavated a suitable cavity after multiple failed attempts. Therefore, we suggest that researchers aiming to measure nest tree quality distinguish frequently-excavated trees that may contain test holes and unfinished cavities from those that are re-used for nesting.

Our finding of distinct nest site choices of earlier breeding pairs is consistent with our prediction that earlier breeders exclude later breeders from making preferred choices due to an ideal despotic distribution, and provides support for our argument that habitat quality may be best defined from the choices of earlier breeders, since these are more likely to be experienced pairs able to reproduce successfully [14, 15]. Our results likely reveal birch as a secondarily chosen resource, once choices were made for aspen as nest trees. The strong positive relationship we found between birch and nest density only existed across patches of old forest where aspen trees preferred for nesting were not rare (> 15 trees per ha), which may explain why previous tests found no correlation between densities of birch trees and nests [26]. Because aspen and birch densities were inversely related, yellow-bellied sapsuckers probably rarely encountered areas with high densities of both trees. These patterns suggest that some earlier-nesting pairs were able to establish territories at these relatively rare sites, which contained an adequate density of preferred aspen trees while also offering a relatively high density of live birch trees. Most later-nesting pairs apparently ignored remaining sites with birch because these contained too few preferred aspen trees.

Our finding of highest nest density in mixedwood forest contradicts results of the use-availability analysis, which showed no selection for mixedwood forest when nest sites were compared to nearby available sites. This finding highlights the importance of considering spatial scale when interpreting results of use-availability studies to measure habitat selection. Because we chose available habitat 250–350 m from nest sites, a high proportion of available unused sites were also in mixedwood forest. The spatial scale of the use-availability analysis was too small for measuring selection among forest types. This finding points to the importance of applying multiple measures of habitat selection beyond reliance on a use-availability design, and to ensure that results are interpreted at the appropriate spatial scale.

The main limitation of our study is that we did not collect data on parental quality or dominance, and instead relied on the assumption that earlier-hatching nests belonged to parents that settled territories earlier and were therefore able to select preferred habitat. We suggest that researchers directly measure the ability of pairs to preclude others from preferred habitat when measuring habitat quality of territorial species. We also acknowledge the lower productivity of later pairs may have been simply due to a declining trend in productivity due to seasonal timing *per se*, rather than the ‘quality’ of pairs. If so, the choices of earlier breeding pairs may not have reflected preferred habitat. The ‘quality’ versus ‘timing’ hypotheses are not mutually exclusive, and there is evidence that both drive the common pattern of seasonal decline in productivity among bird species [58, 59]. More importantly, evidence supporting the timing hypothesis also supports the quality hypothesis [59]. Therefore, we argue this debate should not negate preference being defined by the choices of earlier-breeding or otherwise higher quality pairs that result in higher reproductive performance.

### Toward a conceptual model of woodpecker productivity in relation to tree decay

Cavity size and depth, and thus woodpecker productivity, are likely influenced by the architecture of internal decay in aspen trees, because woodpeckers minimize time-consuming excavation of sapwood by primarily excavating decayed wood [36, 54]. Given that predation is the main cause of nest loss in woodpeckers, and larger mammalian predators can more easily access cavities in sapwood softened by decay, then woodpeckers are likely selecting nest trees with decay compartments of a suitable size ideally surrounded by healthy sapwood [40, 50]. This could explain our finding and that of [11] that more reproductively successful sapsuckers selected less decayed aspen trees. Not only are these trees less prone to breakage, but perhaps these trees also contain ‘sound’ decay compartments in which woodpeckers can excavate suitably sized cavities surrounded by protective hard wood.

The distribution of internal decay in aspen trees has been hypothesized as the main factor explaining excavation efficiency, which may be an important factor explaining the orientation and height of cavity entrances [36, 54, 60, 61]. In addition to excavation efficiency and cavity placement, we hypothesize that the distribution of decay in aspen trees also influences cavity size and depth, and thus breeding productivity. Internal decay in aspen trees varies with distance from the ground and orientation of the outer trunk [36, 37, 54]. Fungal infection in aspen trees primarily originates from the base of the trunk and thus decayed wood ('heart rot) is mostly (70%) found in the lower 5 m [37]. Infection also enters higher up the trunk through branch stubs, which aspen trees compartmentalize into decay pockets surrounded by healthy wood [37, 54]. The volume of decay is found more on the south side of the trunk [36]. Thus, decay is usually found in large southward-oriented columns in the lower part of the tree, but more often in southward-oriented compartments higher in the tree, which are irregularly-shaped and variably-sized dependent on time since infection [36, 37, 54, 60]. So far no studies have investigated how the distribution of decay compartments in aspen trees may influence the placement of cavity entrances and cavity size and depth, while measuring subsequent effects on the breeding productivity of woodpeckers. Decay distribution may explain inconsistent effects of cavity orientation and height on woodpecker productivity [28, 52, 62], since under the ‘decay architecture’ hypothesis cavity height and orientation are secondary characteristics driven by decay and not directly chosen by excavators.

We encourage researchers to test this hypothesis, which would require longitudinal sectioning of aspen trees to determine the location and size of distinct decay compartments. Given that the volume of decay in aspen trees is predicted from site conditions, tree age, and the number of fungal conks [63], it may also be possible to derive predictors of the distribution of decay compartments suitable for woodpeckers. Such variables may be more predictive of nest tree selection, cavity placement, and breeding productivity than variables that are less correlated with the distribution of decay compartments.

## Conclusions

Higher reproductive performance of earlier breeding pairs has been commonly observed among woodpecker species [11, 39, 53]. Our study is the first to show that earlier breeders make habitat choices distinct from those identified from use-availability analysis to measure nest site selection, and that their distinct choices predict their higher performance. Our results show how researchers relying on use-availability analysis to measure habitat preference can fall into an ‘ecological trap’ when attributing incongruence between woodpecker breeding productivity and nest site selection to non-ideal habitat selection. We found that use-availability analysis was useful to identify nest site characteristics important to the breeding population as a whole, but was inadequate for determining vegetation characteristics related to reproductive performance. More generally, we found that vegetation frequently selected for nest sites among the whole breeding population masked distinct selections made by the more reproductively successful portion of the population. By distinguishing the choices of earlier breeding pairs, we identified characteristics of nest sites masked by selection frequency that likely promote lifetime productivity, including abundant food resources and nest trees more resilient to breakage. Identifying distinct choices of earlier breeders for abundant food in the form of birch sap improved our ability to explain the spatial distribution of nests. Further, though only 31% of yellow-bellied sapsucker cavity entrances faced north, it was the more frequent choice of earlier breeders to excavate northward facing cavities that resulted in the highest productivity. We encourage researchers attempting to quantify avian habitat quality to measure the choices of earlier breeding, or otherwise dominant or more experienced pairs.

We also encourage researchers to relate woodpecker productivity to the distribution of internal decay in aspen trees. The results are likely to show that easily-accessed decay compartments of a suitable size and surrounded by healthy sapwood are an important measure of habitat quality. More refined measures of habitat quality will be particularly useful for keystone species like the yellow-bellied sapsucker, for which improved management prescriptions will help sustain species like bats and flying squirrels that rely on the sapwells and cavities they create.

Our results also show the importance of avoiding simple prescriptions for nest trees of a certain type when setting management targets for retention of high quality habitat for woodpeckers. We found evidence that reproductively successful yellow-bellied sapsuckers chose to nest in less decayed aspen trees more resilient to breakage that were within patches of similar trees, likely to ensure future options for nesting, and secured nest sites with abundant food resources in the form of birch sap. Given high rates of trunk breakage and the widespread importance of aspen trees for cavity-using species, management plans need to ensure future recruitment of patches of aspen trees of suitable size and decay, in addition to food resources, particularly for territorial woodpeckers like yellow-bellied sapsuckers showing fidelity to breeding sites [21, 64, 65, 66].

## Supporting information

S1 Data Raw data. (XLSX)

S1 Table Model selection results for analysis of nest site selection of yellow-bellied sapsuckers using conditional logistic regression (n = 56 nest sites). (DOCX)

S2 Table Model selection results of the analysis comparing nest sites of earlier with later breeding yellow-bellied sapsuckers (n = 58 pairs) using logistic regression. (DOCX)

S3 Table Model selection results of the analysis relating fledgling production of yellow-bellied sapsuckers (n = 58 pairs) with nest site characteristics using ordinal logistic regression. (DOCX)

S4 Table Model selection results of the analysis relating the nest density (nests/ha) of yellow-bellied sapsuckers in relation to % deciduous and the density of aspen trees for nesting and live birch trees for food (trees/0.04 ha) using GLM with Poisson error and log link (n = 87 forest patches varying in % deciduous). (DOCX)
