# Supplementary material for: Early breeders choose differently – Refining measures of habitat quality for the yellow-bellied sapsucker (Sphyrapicus varius), a keystone species in the mixedwood boreal forest
Source: PLoS One. 2018 Sep 12;13(9):e0203683. doi: 10.1371/journal.pone.0203683 (PMC6135400; doi:10.1371/journal.pone.0203683)
Supplement: S1 Table — (DOCX) [file pone.0203683.s002.docx]

S1 Table. Model selection results for analysis of nest site selection of yellow-bellied sapsuckers using conditional logistic regression (n=56 nest sites). Models are ranked by difference in Akaike’s Information Criterion corrected for small sample sizes (ΔAICc) from the model with the lowest AICc.

| Scale | Model | K | Log Lik | AICc | ∆AICc | Model weight |
| --- | --- | --- | --- | --- | --- | --- |
| Tree, nest site | Dbh^2^ + conks + live decaying aspen^a^ | 4 | −9.84 | 28.1 | 0 | 0.26 |
| Tree | Dbh^2^ + conks | 3 | −11.5 | 29.3 | 1.2 | 0.14 |
| Tree, territory core | Dbh^2^ + conks + live decaying aspen | 4 | −11.1 | 30.6 | 2.5 | 0.07 |
| Tree, nest site, stand | Dbh^2^ + conks + live decaying aspen + % deciduous | 5 | −9.80 | 30.8 | 2.7 | 0.07 |
| Tree, nest site | Dbh + conks + live decaying aspen | 3 | −12.5 | 31.1 | 3.0 | 0.05 |
| Tree | Dbh^2^ * conks | 4 | −11.4 | 31.2 | 3.1 | 0.05 |
| Tree, nest site, territory core | Dbh^2^+ conks + live decaying aspen (nest site) + birch + shrubs | 6 | −9.27 | 31.3 | 3.2 | 0.05 |
| Tree, nest site, territory core | Dbh^2^+ conks + live decaying aspen (nest site) + birch + shrubs | 6 | −9.27 | 31.3 | 3.2 | 0.05 |
| Tree, territory core | Dbh^2^ + conks + birch + shrubs | 5 | −10.9 | 32.3 | 4.2 | 0.03 |
| Tree, nest site, stand | Dbh^2^ + conks + live decaying aspen + % deciduous^2^ | 6 | -9.36 | 32.4 | 4.3 | 0.03 |
| Tree, nest site, territory core | Dbh + conks + live decaying aspen (nest site) + birch + shrubs | 5 | −11.3 | 33.1 | 5.0 | 0.02 |
| Tree, nest site, stand | Dbh + conks + live decaying aspen + % deciduous | 4 | −12.3 | 33.4 | 5.3 | 0.02 |
| Tree | Dbh + conks | 2 | −14.7 | 33.5 | 5.4 | 0.02 |
| Tree, territory core | Dbh^2^ + conks + birch + shrubs + live decaying aspen | 5 | −10.5 | 33.7 | 5.6 | 0.02 |
| Tree, territory core, stand | Dbh + conks + birch + shrubs + live decaying aspen + % deciduous | 6 | −10.1 | 33.8 | 5.7 | 0.02 |
| Tree, territory core, stand | Dbh^2^ + conks + birch + shrubs + live decaying aspen + % deciduous | 7 | −8.83 | 34.0 | 5.9 | 0.01 |
| Tree | Dbh * conks | 3 | −13.9 | 34.0 | 5.9 | 0.01 |
| Tree, territory core | Dbh + conks + birch + shrubs | 4 | −13.0 | 34.4 | 6.3 | 0.01 |
| Tree, territory core | Dbh + conks + live decaying aspen | 3 | −14.2 | 34.5 | 6.4 | 0.01 |
| Tree, nest site, stand | Dbh + conks + live decaying aspen + % deciduous^2^ | 5 | −11.8 | 34.8 | 6.7 | 0.01 |
| Tree, territory core | Dbh + conks + birch + shrubs + live decaying aspen | 5 | −12.7 | 35.9 | 7.8 | 0 |
| Tree, nest site, stand | Dbh + conks + birch + shrubs + live decaying aspen + % deciduous^2^ | 7 | −10.1 | 36.5 | 8.4 | 0 |
| Tree, nest site, stand | Dbh^2^ + conks + birch + shrubs + live decaying aspen + % deciduous^2^ | 8 | −8.82 | 36.7 | 8.6 | 0 |
| Tree | Dbh^2^ | 2 | −18.7 | 41.5 | 13.4 | 0 |
| Tree | Dbh | 1 | −23.7 | 49.5 | 21.4 | 0 |
| Tree | Conks | 1 | −27.1 | 56.2 | 28.1 | 0 |
| Stand | % deciduous^2^ | 2 | −28.9 | 61.8 | 33.7 | 0 |
| Nest site | Live decaying aspen | 1 | −32.3 | 66.7 | 38.6 | 0 |
| Stand | % deciduous | 1 | -32.4 | 66.9 | 38.8 | 0 |
| Territory core | Live decaying aspen | 1 | −35.6 | 73.4 | 45.3 | 0 |
| Territory core | Birch + shrubs + live decaying aspen | 3 | −35.4 | 77.0 | 48.9 | 0 |
| Territory core | Birch + shrubs | 2 | −38.5 | 81.1 | 53.0 | 0 |

^a^ 22-52 cm dbh
