# Supplementary material for: Early breeders choose differently – Refining measures of habitat quality for the yellow-bellied sapsucker (Sphyrapicus varius), a keystone species in the mixedwood boreal forest
Source: PLoS One. 2018 Sep 12;13(9):e0203683. doi: 10.1371/journal.pone.0203683 (PMC6135400; doi:10.1371/journal.pone.0203683)
Supplement: S2 Table — (DOCX) [file pone.0203683.s003.docx]

Table S2. Model selection results of the analysis comparing nest sites of earlier with later breeding yellow-bellied sapsuckers (n=58 pairs) using logistic regression. Models are ranked by difference in Akaike’s Information Criterion corrected for small sample sizes (ΔAICc) from the model with the lowest AICc.

| Scale | Model | K | Log likelihood | AICc | ∆AICc | Model weight |
| --- | --- | --- | --- | --- | --- | --- |
| Cavity, tree, nest site | Cavity height + conks + birch | 4 | −31.4 | 71.5 | 0 | 0.15 |
| Cavity, tree, nest site | Cavity aspect + conks + birch | 4 | −31.7 | 72.1 | 0.6 | 0.11 |
| Cavity, territory | Cavity height + cavity aspect + birch + shrub | 5 | −30.8 | 72.7 | 1.2 | 0.08 |
| Cavity, tree, nest site | Cavity height + dbh + conks + birch | 5 | −31.4 | 74.0 | 2.5 | 0.04 |
| Cavity, tree, nest site, territory core, stand | Cavity height + cavity aspect + dbh + conks + live decaying aspen (nest site) + birch (territory) + shrubs (territory) + % deciduous | 9 | -26.2 | 74.2 | 2.7 | 0.04 |
| Cavity, territory core | Cavity height + cavity aspect + birch + live decaying aspen | 5 | −31.6 | 74.3 | 2.8 | 0.04 |
| Cavity, tree, nest site, territory core, stand | Cavity height + cavity aspect + dbh^2^ + conks + live decaying aspen (nest site) + birch (territory) + shrubs (territory) + % deciduous | 10 | -24.8 | 74.3 | 2.8 | 0.04 |
| Territory, stand | Shrubs + birch + % deciduous | 4 | -32.8 | 74.4 | 2.9 | 0.04 |
| Cavity, tree, nest site | Cavity aspect + dbh + conks + birch | 5 | −31.7 | 74.5 | 3.0 | 0.03 |
| Cavity, tree, nest site | Cavity height + cavity aspect + dbh + conks+ birch | 6 | −30.5 | 74.6 | 3.1 | 0.03 |
| Cavity, tree, nest site | Cavity height + dbh^2^ + conks + birch | 6 | −30.6 | 74.8 | 3.3 | 0.03 |
| Tree, nest site | Dbh + conks + birch | 4 | −33.1 | 74.9 | 3.4 | 0.03 |
| Cavity, tree | Cavity height + cavity aspect + conks | 4 | −33.1 | 75.0 | 3.5 | 0.03 |
| Cavity, tree, nest site | Cavity height + cavity aspect + dbh^2^ + conks+ birch | 7 | −29.5 | 75.2 | 3.7 | 0.02 |
| Cavity, tree, nest site | Cavity aspect + dbh^2^ + conks + birch | 6 | −30.8 | 75.3 | 3.8 | 0.02 |
| Cavity | Cavity aspect | 2 | −35.6 | 75.3 | 3.8 | 0.02 |
| Tree, territory, stand | Dbh + conks + shrubs + birch + % deciduous | 6 | −31.0 | 75.8 | 4.3 | 0.02 |
| Tree, nest site | Dbh^2^ + conks + birch | 5 | −32.5 | 76.1 | 4.6 | 0.02 |
| Territory, stand | Shrubs + birch + % deciduous^2^ | 5 | −32.5 | 76.1 | 4.6 | 0.02 |
| Cavity | Cavity height + aspect | 3 | −34.9 | 76.3 | 4.8 | 0.01 |
| Cavity, tree, nest site | Cavity height + conks + live decaying aspen | 4 | −33.8 | 76.4 | 4.9 | 0.01 |
| Cavity, tree, nest site | Cavity aspect + conks + live decaying aspen^a^ | 4 | −33.8 | 76.4 | 4.9 | 0.01 |
| Cavity, tree | Cavity height + cavity aspect + dbh^2^ + conks | 6 | −31.4 | 76.5 | 5 | 0.01 |
| Cavity, tree, nest site, territory core, stand | Cavity height + cavity aspect + dbh + conks + live decaying aspen (nest site) + birch (territory) + shrubs (territory) + % deciduous^2^ | 10 | -25.9 | 76.6 | 5.1 | 0.01 |
| Cavity, tree | Cavity height + cavity aspect + dbh + conks | 5 | −33.0 | 77.1 | 5.6 | 0.01 |
| Stand | % deciduous | 2 | -36.5 | 77.2 | 5.7 | 0.01 |
| Territory | Shrubs + birch + live decaying aspen | 4 | −34.4 | 77.5 | 6.0 | 0.01 |
| Tree | Dbh + conks + cavity count | 4 | −34.4 | 77.7 | 6.2 | 0.01 |
| Cavity, tree, nest site, territory core | Cavity height + cavity aspect + dbh + conks + live decaying aspen^a^ (nest site) + birch (territory) + shrubs (territory) | 8 | −29.3 | 77.7 | 6.2 | 0.01 |
| Tree | Dbh + conks | 3 | −35.6 | 77.8 | 6.3 | 0.01 |
| Tree, territory, stand | Dbh + conks + shrubs + birch + % deciduous^2^ | 7 | -30.8 | 77.9 | 6.4 | 0.01 |
| Stand | % deciduous^2^ | 3 | -35.8 | 78 | 6.5 | 0.01 |
| Tree | Dbh^2^ + conks + cavity count | 5 | −33.4 | 78.1 | 6.6 | 0.01 |
| Cavity | Cavity height | 2 | −37.0 | 78.2 | 6.7 | 0.01 |
| Tree | Dbh^2^ + conks | 4 | −34.8 | 78.3 | 6.8 | 0.01 |
| Cavity, tree, nest site, territory core | Cavity height + cavity aspect + dbh^2^+ conks + live decaying aspen^a^ (nest site) + birch (territory) + shrubs (territory) | 9 | −28.3 | 78.3 | 6.8 | 0.01 |
| Nest site, territory | Live decaying aspen (nest site) + shrubs + birch | 4 | −34.8 | 78.3 | 6.8 | 0.01 |
| Cavity, territory | Cavity height + cavity aspect + live decaying aspen | 4 | −34.8 | 78.4 | 6.9 | 0 |
| Cavity, tree, nest site | Cavity height + cavity aspect + dbh^2^ + conks+ live decaying aspen | 7 | −31.1 | 78.6 | 7.1 | 0 |
| Cavity, tree, nest site | Cavity height + cavity aspect + dbh + conks+ live decaying aspen | 7 | −31.1 | 78.6 | 7.1 | 0 |
| Cavity, tree, nest site, territory core, stand | Cavity height^2^ + cavity aspect + dbh + conks + live decaying aspen (nest site) + birch (territory) + shrubs (territory) + % deciduous^2^ | 11 | -25.4 | 78.6 | 7.1 | 0 |
| Cavity, tree, nest site | Cavity aspect + dbh^2^ + conks + live decaying aspen | 6 | −32.5 | 78.8 | 7.3 | 0 |
| Cavity, tree, nest site | Cavity aspect + dbh + conks + live decaying aspen | 5 | −33.8 | 78.8 | 7.3 | 0 |
| Cavity, tree, nest site | Cavity height + dbh + conks + live decaying aspen | 5 | −33.8 | 78.8 | 7.3 | 0 |
| Cavity, tree, nest site | Cavity height + dbh^2^ + conks + live decaying aspen | 6 | −32.5 | 78.8 | 7.3 | 0 |
| Tree, nest site, territory | Dbh + conks + live decaying aspen (nest site) + birch + shrubs | 7 | −32.6 | 78.8 | 7.3 | 0 |
| Null |  | 1 | −38.5 | 79 | 7.5 | 0 |
| Nest site selection | Dbh^2^ + conks + live decaying aspen | 6 | −32.7 | 79.1 | 7.6 | 0 |
| Tree, territory, stand | Dbh^2^ + conks + shrubs + birch + % deciduous | 7 | -30.2 | 79.6 | 8.1 | 0 |
| Tree, territory, stand | Dbh^2^ + conks + shrubs + birch + % deciduous^2^ | 8 | −30.5 | 80 | 8.5 | 0 |
| Tree, nest site, territory | Dbh^2^ + conks + live decaying aspen (nest site) + birch + shrubs | 7 | −32.0 | 80.3 | 8.8 | 0 |
| Nest site | Live decaying aspen | 2 | −38.2 | 80.6 | 9.1 | 0 |

^a^22-52 cm dbh
