# Supplementary material for: Early breeders choose differently – Refining measures of habitat quality for the yellow-bellied sapsucker (Sphyrapicus varius), a keystone species in the mixedwood boreal forest
Source: PLoS One. 2018 Sep 12;13(9):e0203683. doi: 10.1371/journal.pone.0203683 (PMC6135400; doi:10.1371/journal.pone.0203683)
Supplement: S3 Table — (DOCX) [file pone.0203683.s004.docx]

Table S3. Model selection results of the analysis relating fledgling production of yellow-bellied sapsuckers (n=58 pairs) with nest site characteristics using ordinal logistic regression. Models are ranked by difference in Akaike’s Information Criterion corrected for small sample sizes (ΔAICc) from the model with the lowest AICc.

| Scale | Model | K | Log likelihood | AICc | ∆AICc | Model weight |
| --- | --- | --- | --- | --- | --- | --- |
| Timing + cavity | Hatching date, cavity aspect, cavity height | 5 | −50.6 | 112.3 | 0 | 0.61 |
| Timing only | Hatching date | 3 | −54.3 | 115 | 2.7 | 0.16 |
| Timing, territory | Hatching date + live decaying aspen + shrub + birch | 6 | −52.0 | 117.7 | 5.4 | 0.04 |
| Timing, cavity, tree | Hatching date + dbh + conks + cavity count + cavity height + cavity aspect | 8 | −49.7 | 118.3 | 6.0 | 0.03 |
| Timing, territory, stand | Hatching date + birch + shrubs + % deciduous | 6 | −51.8 | 117.3 | 6.2 | 0.03 |
| Timing, cavity, tree, nest site | Hatching date + dbh + conks + cavity height + cavity aspect + live decaying aspen^a^ | 8 | −50.2 | 119.2 | 6.9 | 0.02 |
| Timing, neighbourhood, stand | Year + hatching date + area + % deciduous^2^ | 7 | −52.5 | 119.5 | 7.2 | 0.02 |
| Timing, neighbourhood, stand | Year + hatching date + area + % deciduous | 6 | −52.8 | 119.2 | 7.2 | 0.02 |
| Cavity | Cavity aspect + height | 4 | −55.4 | 119.6 | 7.3 | 0.02 |
| Timing, cavity, tree | Hatching date + dbh + conks + cavity count + cavity height + cavity aspect | 9 | −49.3 | 120.3 | 8.0 | 0.01 |
| Timing, tree | Hatching date + dbh + conks + cavity count | 6 | −53.4 | 120.5 | 8.2 | 0.01 |
| Timing, territory, stand | Hatching date + birch + shrubs + % deciduous^2^ | 7 | −51.5 | 119.3 | 8.8 | 0.01 |
| Timing, cavity, tree | Hatching date + dbh^2^ + conks + cavity count + cavity height + cavity aspect | 9 | −49.9 | 121.5 | 9.2 | 0.01 |
| Timing, tree, territory | Hatching date + dbh + conks + live decaying aspen + shrubs + birch | 9 | −51.3 | 121.5 | 9.2 | 0.01 |
| Timing, tree, territory | Hatching date + dbh + conks + cavity count + shrubs + birch | 8 | −51.5 | 122 | 9.7 | 0 |
| Earlier breeding | Conks + cavity height + cavity aspect + birch | 7 | −54.4 | 122.4 | 10.1 | 0 |
| Timing, tree | Hatching date + dbh^2^ + conks + cavity count | 7 | −53.2 | 122.6 | 10.3 | 0 |
| Timing, tree, territory | Hatching date + dbh^2^ + conks + live decaying aspen + shrubs + birch | 10 | −60.0 | 123.7 | 11.4 | 0 |
| Territory | Shrubs + birch | 4 | −57.7 | 124.1 | 11.8 | 0 |
| Timing, tree, territory | Hatching date + dbh^2^ + conks + cavity count + shrubs + birch | 9 | −51.1 | 124.1 | 11.8 | 0 |
| Null |  | 2 | −60.2 | 124.7 | 12.4 | 0 |
| Timing, cavity, tree, nest site, territory, stand | Year + hatching date + cavity height + cavity aspect + dbh + conks + live decaying aspen (nest site) + birch + shrubs + % deciduous | 12 | −47.0 | 128.5 | 13.4 | 0 |
|  | Year | 2 | −60.1 | 126.6 | 14.3 | 0 |
| Neighbourhood | Location | 3 | −60.2 | 126.9 | 14.6 | 0 |
| Stand | % deciduous | 3 | −60.2 | 126.9 | 14.6 | 0 |
| Timing, cavity, tree, nest site, territory, stand | Year + hatching date + cavity height + cavity aspect + dbh + conks + live decaying aspen (nest site) + birch + shrubs + % deciduous^2^ | 13 | −47.2 | 125.4 | 16.2 | 0 |
| Stand | % deciduous^2^ | 4 | -59.8 | 128.4 | 16.3 | 0 |
| Tree | Dbh + conks + cavity count | 5 | −59.0 | 129.1 | 16.8 | 0 |
| Nest site selection | Dbh^2^ + conks + live decaying aspen (nest site) | 7 | −58.8 | 131.3 | 19.0 | 0 |
| Tree | Dbh^2^ + conks + cavity count | 6 | −59.0 | 131.6 | 19.3 | 0 |

^a^ 22-52 cm dbh
