# Supplementary material for: Early breeders choose differently – Refining measures of habitat quality for the yellow-bellied sapsucker (Sphyrapicus varius), a keystone species in the mixedwood boreal forest
Source: PLoS One. 2018 Sep 12;13(9):e0203683. doi: 10.1371/journal.pone.0203683 (PMC6135400; doi:10.1371/journal.pone.0203683)
Supplement: S4 Table — (DOCX) [file pone.0203683.s005.docx]

Table S4. Model selection results of the analysis relating the nest density (nests/ha) of yellow-bellied sapsuckers in relation to % deciduous and the density of aspen trees for nesting and live birch trees for food (trees/0.04 ha) using GLM with Poisson error and log link (n=87 stands). Models are ranked by difference in Akaike’s Information Criterion corrected for small sample sizes (ΔAICc) from the model with the lowest AICc.

| Model | K | Log likelihood | AICc | ∆AICc | Model weight |
| --- | --- | --- | --- | --- | --- |
| Deciduous^2^ + birch*live decaying aspen^a^ | 6 | −89.0 | 191.0 | 0 | 0.44 |
| Deciduous^2^ + birch | 4 | −91.5 | 191.5 | 0.50 | 0.34 |
| Deciduous^2^ + birch + live decaying aspen | 5 | −91.3 | 193.3 | 2.30 | 0.14 |
| Deciduous + birch*live decaying aspen | 5 | −93.2 | 197.2 | 6.20 | 0.02 |
| Deciduous + birch | 3 | −95.8 | 197.8 | 6.80 | 0.01 |
| Deciduous^2^ | 3 | −95.9 | 198.2 | 7.20 | 0.01 |
| Deciduous + birch + live decaying aspen | 4 | −95.1 | 198.7 | 7.70 | 0.01 |
| Deciduous*live decaying aspen + birch | 5 | −94.5 | 199.8 | 8.80 | 0.00 |
| Live decaying aspen * birch | 4 | −95.8 | 200.1 | 9.10 | 0.00 |
| Deciduous*birch | 4 | −95.8 | 200.2 | 9.20 | 0.00 |
| Deciduous^2^ + live decaying aspen | 4 | −95.9 | 200.4 | 9.40 | 0.00 |
| Deciduous*birch + live decaying aspen | 5 | −95.1 | 201.0 | 10.0 | 0.00 |
| Live decaying aspen + birch | 3 | −98.1 | 202.6 | 11.6 | 0.00 |
| Birch | 2 | −101 | 205.3 | 14.3 | 0.00 |
| Deciduous | 2 | −102 | 207.9 | 16.9 | 0.00 |
| Deciduous + live decaying aspen | 3 | −102 | 209.6 | 18.6 | 0.00 |
| Deciduous*live decaying aspen | 4 | −101 | 210.6 | 19.6 | 0.00 |
| Live decaying aspen | 2 | −106 | 216.1 | 25.1 | 0.00 |
| Null | 1 | −107 | 216.9 | 25.9 | 0.00 |

^a^ 22-41 cm dbh
